# 

**Published:** 1859-12

**Authors:** 


☞ Prof. Austin Flint has now left New York, to enter upon the duties of his Chair in the New Orleans School of Medicine. He will return early next spring; and, until then, correspondents will please address him at New Orleans.

Our office and residence is still at 111 East 21st street, Gramercy-Park House.

